# Normative Data for a Rapid, Automated Test of Spatial Release From Masking

**DOI:** 10.1044/2018_AJA-17-0069

**Published:** 2018-12-06

**Authors:** Kasey M. Jakien, Frederick J. Gallun

**Affiliations:** a National Center for Rehabilitative Auditory Research, VA Portland Health Care System, Department of Veterans Affairs, OR; b Department of Otolaryngology–Head & Neck Surgery, Oregon Health and Science University, Portland

## Abstract

**Purpose:**

The purpose of this study is to report normative data and predict thresholds for a rapid test of spatial release from masking for speech perception. The test is easily administered and has good repeatability, with the potential to be used in clinics and laboratories. Normative functions were generated for adults varying in age and amounts of hearing loss.

**Method:**

The test of spatial release presents a virtual auditory scene over headphones with 2 conditions: colocated (with target and maskers at 0°) and spatially separated (with target at 0° and maskers at ± 45°). Listener thresholds are determined as target-to-masker ratios, and spatial release from masking (SRM) is determined as the difference between the colocated condition and spatially separated condition. Multiple linear regression was used to fit the data from 82 adults 18–80 years of age with normal to moderate hearing loss (0–40 dB HL pure-tone average [PTA]). The regression equations were then used to generate normative functions that relate age (in years) and hearing thresholds (as PTA) to target-to-masker ratios and SRM.

**Results:**

Normative functions were able to predict thresholds with an error of less than 3.5 dB in all conditions. In the colocated condition, the function included only age as a predictive parameter, whereas in the spatially separated condition, both age and PTA were included as parameters. For SRM, PTA was the only significant predictor. Different functions were generated for the 1st run, the 2nd run, and the average of the 2 runs. All 3 functions were largely similar in form, with the smallest error being associated with the function on the basis of the average of 2 runs.

**Conclusion:**

With the normative functions generated from this data set, it would be possible for a researcher or clinician to interpret data from a small number of participants or even a single patient without having to first collect data from a control group, substantially reducing the time and resources needed.

**Supplemental Material:**

https://doi.org/10.23641/asha.7080878

Our goal is to report normative data and predict thresholds for a rapid test of spatial release from masking (SRM) for speech perception. SRM refers to the benefit that a listener obtains when a target sound source is at a different location in space relative to one or more masking sound sources. This benefit is reflected in the ability to understand speech in adverse environments with maskers that are much more intense than the target. Performance improvement depends on the presence of interaural differences in both time and level, both of which are less effective for listeners with increasing age and hearing loss (Ellinger, Jakien, & Gallun, 2017). However, the audiogram is not a sufficient predictor of who may struggle to achieve SRM (Ruggles & Shinn-Cunningham, 2011; Strelcyk & Dau, 2009). For this reason, reduced SRM abilities have also been suggested to represent a form of central auditory processing disorder (Cameron & Dillon, 2008; Cameron, Glyde, & Dillon, 2011), for which clinical tests have been developed, the Listening in Spatialized Noise–Sentences test (LISN-S) and the North American Listening in Spatialized Noise–Sentences test (NA LiSN-S; Cameron et al., 2009; Cameron & Dillon, 2007), that allow clinicians to compare performance of patients to normative standards on the use of both spatial and voice cues. A successful test of SRM to potentially diagnose central auditory processing disorder must be reliable (Cameron et al., 2009; Jakien, Gallun, Stansell, & Kampel, 2017) and represent a large breadth of the population, so as to be most useful for clinical implementation (Cameron et al., 2011). In order to be most useful clinically, however, a new clinical test should also require as few clinical resources as possible and, like the audiogram, have high test–retest reliability. While representing a large population and obtaining good reliability, the LISN requires an administrator to listen as the patient repeats back sentences and, then, score the keywords as correct or incorrect. This both increases the resources needed to administer the test and introduces a potential source of error in that now there are two people who must pay attention and make responses in order for performance to be measured accurately. This additional resource requirement is a potential barrier to the use of these tests in the clinic, where time and resources are always limited. Thus, it would be useful to have a rapid and automated test of SRM that takes approximately 5 to 7 min to complete to save clinician time and effort.

Another aspect of clinical utility is the ability to be used for repeated testing. The LISN has a limited set of open-set materials that cannot be repeated an arbitrary number of times without running the risk of participants learning the materials, making it unsuitable for monitoring changes in performance across a large number of time points. Westermann and Buchholz (2015) demonstrated that the benefits of changes in perceived distance between targets and maskers for the LISN are similar to that found with the Coordinate Response Measure Corpus (CRM; Bolia, Nelson, Ericson, & Simpson, 2000). The CRM is a closed set test that can be easily automated and repeated multiple times with only minimal improvements in performance (Jakien et al., 2017). In addition, Humes, Kidd, and Fogerty (2017) have recently established that tests using the CRM are consistent with more traditional tests of speech perception in multitalker babble in terms of performance both as a function of target-to-masker ratio (TMR) and of percent correct. The CRM has been utilized in over 50 studies to assess speech intelligibility across a wide range of listening situations (Arbogast, Mason, & Kidd, 2002; Best, Gallun, Ihlefeld, & Shinn-Cunningham, 2006; Brungart, 2001; Humes, Lee, & Coughlin, 2006; Ihlefeld & Shinn-Cunningham, 2008; Johnsrude et al., 2013; Mesgarani & Chang, 2012; Rossi-Katz & Arehart, 2009; Singh, Pichora-Fuller, & Schneider, 2008) and has been shown to have the psychometric properties necessary for use in a both fixed-level and adaptive-level psychometric procedures across different types of speech and noise maskers (Eddins & Liu, 2012).

The analyses presented below describe the calculation of normative functions for an automated CRM-based test of SRM (the SR2, which stands for “spatial release with two maskers”), which was developed by Marrone, Mason, and Kidd (2008a) and modified for rapid testing using a progressive tracking procedure by Gallun, Diedesch, Kampel, and Jakien (2013). It is also very similar to the configuration tested by Westermann and Buchholz (2015), with the present study using horizontal separation instead of distance to achieve spatial separation. The automated test simultaneously presents three speech sentences (one target and two maskers) at one of two spatial configurations: colocated (with the target sentence and masking sentences at 0°) or spatially separated (with the target at 0° and the maskers at ±45°). Listener thresholds are documented as TMRs, and SRM is determined as the difference between the colocated and spatially separated conditions. A detailed description of the development of the rapid version of the SR2 can be found in Gallun et al. (2013), and the test–retest reliability of the SR2 is shown in Jakien et al. (2017). For the past 5 years, the authors have been sharing the code for the SR2 with other researchers and have recently established a GitHub repository (https://github.com/gallunf/SR2) where anyone interested can download the MATLAB code and the wavefiles needed to run the experiments. Over the past year, in collaboration with the University of California Riverside Brain Game Center and on the basis of a grant from the National Institutes of Health, the authors have also created a version of the SR2 (called *Spatial Release*) to be run on an iPad. Spatial Release is the same program as the SR2, takes only 5 to 7 min to complete, and is available to clinicians and the general public for free on the iTunes app store (https://bgc.ucr.edu/games/spatialrelease/). Currently, multiple sites, both in the Veterans Administration system and at other universities, are using either the SR2 or Spatial Release to collect data on adults. The authors will continue to make the SR2 code available to anyone who is interested and are in the process of establishing the degree to which the SR2 and Spatial Release can be used interchangeably.

Normative data are commonly collected on a sample of listeners with no known deficit and expressed as a cutoff score on the basis of the means and standard deviations of the measures recorded on this sample (American Academy of Audiology, 2010; American Speech-Language-Hearing Association, 2005). Typical cutoff values for tests of auditory processing are 2 or 3 *SD*s below the mean. One difficulty with this approach, as described in detail by Tomlin, Dillon, and Kelly (2014), is that it may be necessary to establish different cutoff values for different members of the normal sample. Those researchers describe the difficulties associated with establishing cutoffs for children varying in age between 7 and 12 years. Similarly, the data reviewed above clearly demonstrate that, even with no diagnosis of an auditory processing deficit, listeners varying in age and hearing ability are expected to vary in the ability to use spatial cues. To address this issue and allow clinicians to assess SRM in patients who may be older and may have impaired hearing, we have adapted the regression method of Tomlin et al. (2014) to a large data set collected at the VA Portland Health Care System.

While examining the effects of age and hearing loss on SRM, it is important to note previous studies that have also examined how age and hearing loss affect SRM for adults (Ahlstrom, Horwitz, & Dubno, 2014; Besser, Festen, Goverts, Kramer, & Pichora-Fuller, 2015; Best, Marrone, Mason, & Kidd, 2012; Dubno, Dirks, & Morgan, 1984; Helfer & Freyman, 2008; Marrone, Mason, & Kidd, 2008b; Srinivasan, Jakien, & Gallun, 2016). Some studies found age effects, others hearing loss effects, and a few found both. One explanation for the divergent findings is that age effects found with a spatial release task can be overshadowed by hearing loss. Gallun et al. (2013) showed strong age effects when participants were recruited such that age and hearing thresholds were weakly related. Marrone et al. (2008b) found strong hearing loss effects in combination with a trend suggesting that aging affects SRM, but the trend failed to reach statistical significance. The results of the work of Srinivasan et al. (2016) were consistent with the suggestion that age effects in an SRM task are mostly easily observed at smaller separations between target and maskers (less than 10°) than are commonly tested, whereas hearing loss dominates performance for separations of 15° and larger, as has been found in studies that only test larger separations (e.g., Glyde, Cameron, Dillon, Hickson, & Seeto, 2013; Jakien et al., 2017; Marrone et al., 2008b). Because many patients in an audiology clinic have some form of hearing loss and are likely to be drawn from a broad range of ages, we believe that it is important to find a method of incorporating both age and hearing loss into normative functions for a test of spatial release. The approach we have adopted involves the use of multiple linear regression in which both age and hearing loss are included as predictive variables.

Below, we present a range of normative values and the regression functions needed to incorporate age, hearing loss, or both into a comparison of SRM for a given listener with expected performance. The resulting functions predict mean and expected ranges of performance on the SR2 for listeners varying in age between 18 and 80 years and four frequency bilateral pure-tone average (PTA) of 0.5-, 1-, 2-, and 4-kHz hearing losses up to 40 dB HL. This work expands the findings of Gallun et al. (2013), which introduced the SR2 and compared it to traditional adaptive tracking methods in both anechoic conditions and under headphones in a virtual display. Gallun et al. (2013) also showed that, when all participants have hearing in the normal range, stronger age effects can be observed. The data presented in Jakien et al. (2017) were an expansion of the Gallun et al. (2013) data to include more listeners and repeated administrations of the SR2 (up to eight repetitions). The repeated testing was added 2 years after the first listeners were tested (data from whom were presented by Gallun et al., 2013) and was opportunistically conducted in conjunction with one of the other experiments being run in the laboratory. For those listeners whose data were reported by Gallun et al. (2013), repeated testing occurred when they returned to do another experiment. For a larger group of listeners, repeated testing had already been implemented when they first visited the laboratory. These participants were often tested within 1 or 2 weeks, and eventually, the testing was even performed at the beginning and end of a single 1-hr test session. As a result, the delay between tests ranged between 1 hr and 3 years. Due to this large variation in delay, the two sessions for each listener are analyzed separately and in combination. Test–retest reliability was examined by Jakien et al. (2017) where it was found that there was improvement in performance from Run 1–2 of the test and little improvement in performance from Runs 2–8. On the basis of this finding, we have analyzed the data from 82 participants who all completed two runs of the SR2, which allows us to use a large sample size while also calculating normative functions on the basis of the first run, the second run, and the average of both runs. The data presented below show that the precision of the normative functions is improved the most by averaging the two runs but that the normative functions show very similar patterns regardless of the subset of the data analyzed. The similarity of the predictions for the first and second run is another way of confirming the test–retest reliability of this data set, another aspect of which was tested by Jakien et al. (2017).

## General Method

### Participants

**Figure 1.**
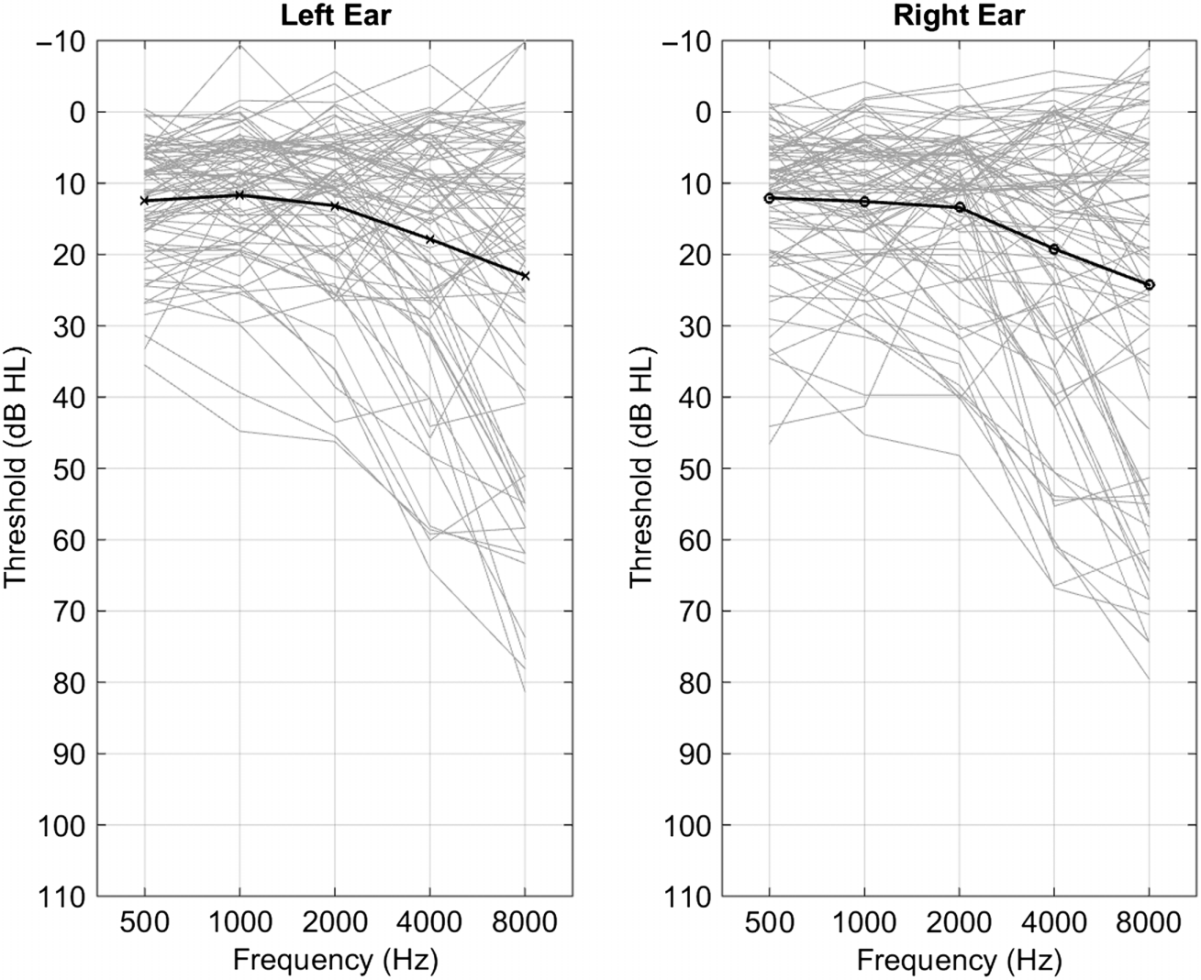
Individual (light lines) and average (dark lines) audiograms for all 82 participants for left and right ears. Individual audiograms are jittered by up to 2 dB for visibility.

Over the past 5 years (2012–2017), 100 participants completed at least one test session involving the SR2, and as described in Jakien et al. (2017), 40 participants completed at least eight repetitions of these procedures. In this report, we have extracted the data from the 82 participants (49 male, 33 female) who completed at least two repetitions. The delay between the two repetitions was longer than a year for 33 of the participants, between a month and a year for 14 participants, between a week and a month for 12 participants, and between a day and week for seven participants, and for 16 participants, both tests and retest occurred on the same day. Figure 1 shows the individual audiograms and the average for the left and right ears for all 82 individuals. The individual functions (light gray lines) are jittered by 2 dB for visual clarity. The mean age was 46.74 years (*SD* = 18.66). Mean bilateral PTA (calculated as the average of 0.5, 1, 2, and 4 kHz across the left and right ears) was 12.48 dB relative to standard hearing level (American National Standards Institute, 2004) with an *SD* of 8.89 dB. Five of the participants reported wearing hearing aids, although the aids were not worn during testing. In order to ensure that audibility could be equated as described below, all participants were required to have PTAs of 45 dB or below. All listeners had fairly symmetrical hearing below 2 kHz: Only 27/82 participants had greater than 10-dB differences at any frequency below 2 kHz, and no participants had differences of greater than 20 dB at any frequency below 2 kHz. Across all audiometric frequencies, only 17/82 participants had differences of 15–20 dB (mostly above 2 kHz), and eight had asymmetries greater than 20 dB (all above 2 kHz). None had differences exceeding 35 dB at any frequency. The full audiogram data for each participant, along with gender, hearing aid use, and calculated values of PTA and asymmetry across ears, are included in the Supplemental Material S1. All listeners were in good health with no history of otological disorders and had scores of 24 or higher on the Mini-Mental State Examination (Folstein et al., 1975) to rule out cognitive impairment. Tympanograms were taken and bone-conduction audiometry was used to ensure that all hearing loss was sensorineural. Procedures were approved by the VA Portland Health Care System Institutional Review Board, and all participants were monetarily compensated for their time.

### Stimuli

As described by Bolia et al. (2000), CRM sentences have the form: “Ready, CALLSIGN, go to COLOR, NUMBER, now,” and for the SR2, three of the four male talkers included in the CRM (Talkers 0, 1, and 2) are used to present the target and masking sentences. Talker 3 was not used due to the talker's slower rate of speaking. Head-related impulse responses from KEMAR recordings from the Center for Image Processing and Integrated Computing database acquired through the Music and Audio Research Laboratory at New York University (Andreopoulou & Roginska, 2011) corresponding to the different spatial locations were convolved with the target and masking speech, resulting in a virtual auditory scene. Etymotic ER2 insert earphones (Etymotic Research) were used, and target sentences were presented at an equal sensation level (SL) of 39.5 dB across all listeners. Equal SL was achieved by first measuring the speech reception threshold (SRT) for each listener using standard audiometric methods and then presenting the target at a fixed level above the SRT of 39.5-dB SL. The two masking sentences were presented at a range of TMR values between −8 dB and 10 dB, which resulted in masking stimuli that were presented at levels between 31.5 dB and 49.5 dB above that individual's SRT. No listeners were tested for whom the maskers exceeded 85 dB SPL.

### Procedure

All testing was done in a sound-attenuated booth at the National Center for Rehabilitative Research in Portland, OR. Digitally recorded speech stimuli were processed in MATLAB (Mathworks) using the convolution described above, converted to analog signals using either a Grace m903 (Grace Design) or Lynx Hilo (Lynx Studio Technology) digital to analog converter and amplifier. Signals were presented over Etymotic ER2 earphones, and participants selected their responses using a graphical display of colored numbers presented in MATLAB on a computer touch screen monitor.

Participants listened for the sentence with the predetermined call sign (in this case, “Charlie”) and input the color–number combination following the call sign. A progressive tracking procedure was implemented to present 20 trials, two at each of 10 TMRs, starting at 10-dB TMR and ending at −8-dB TMR (decreasing in steps of 2 dB). TMR thresholds in dB were estimated by subtracting the number of correct responses from 10. For example, if a listener answered all 20 presentations correctly, the threshold would be estimated at −10 dB, whereas if all of the answers were incorrect, the estimate of TMR at threshold would be 10 dB. A listener who answered half of the presentations correctly would be assigned a threshold of 0-dB TMR. Gallun et al. (2015) applied a Bayesian analysis to the psychometric functions and determined that this approach is most accurate for thresholds between 6-dB and −6-dB TMR and that the greatest errors are in the underestimation of thresholds for listeners with very good performance (better than −6 dB). As the goal of the rapid method is to screen for dysfunction, the gain in speed at the sacrifice of accuracy is appropriate for this use.

### Statistical Approach

Following Tomlin et al. (2014), a regression analysis was used to establish the relative influence of the factors of age and hearing loss on performance. The data were first examined to determine whether or not linear regression with two variables was appropriate (see Results section below), and then, the regression equation was used to generate predictions for the entire data set. The weights from the regression equation (“β” values) and the constant were then combined in a linear function to allow a predicted value to be calculated for any combination of age and hearing loss. The difference between the predictions on the basis of the regression analysis and the observed values (referred to as the “model error”) was used to estimate the variance of the distribution around the predictive functions. This function can then be used to transform any observed value into a Z-score, which indicates the distance between the observed and predicted values in units of standard deviation. This z-transform was applied to these data and used to identify participants with abnormally large deviations from the expected values. According to Cohen and Cohen (1983), a multiple linear regression with two factors and 82 participants has a power of .8 (80% chance of observing the relationship) and a *p* value of .05 as long as the regression explains at least 11% of the variance. For a single predictive factor, the power is .8 if there is at least 9% of the variance explained, power of .7 if 7% is explained, and power of .5 if 5% is explained. This analysis suggests that 82 participants are sufficient to have at least a 50% chance of finding even quite small relationships.

## Results

Table 1 shows mean thresholds in TMR and the difference, expressed as SRM, for each condition for each of the two runs and for the average of both runs, as well as standard deviations. The full data set, along with demographic variables and potential predictors, are available as Supplemental Material S1. Thresholds were reduced from Run 1 to Run 2 by 0.7 dB for the colocated condition and 1.4 dB for separated condition, resulting in an increase of 0.7 dB in SRM. This is consistent with the changes observed for a subset of these participants in Jakien et al. (2017). Table 2 shows the correlations of the SR2 thresholds with age and hearing loss. Hearing loss is characterized as the SRT, the standard PTA (average of 0.5, 1, 2, and 4 kHz), and the high-frequency PTA (average of 1, 2, 4, and 8 kHz). Each is the average of the threshold from the left and right ears (“bilateral”). To adjust for the 15 correlations considered, Bonferroni correction was applied before analysis of significance, which suggested treating a *p* value of .0033 as the criterion for significance, which corresponds to a correlation of .333 or higher. Correlations between age and thresholds in the colocated condition (*r* = .388) were significant, whereas correlations with hearing loss were not (*r* = .274 or lower). Thresholds in the separated condition were correlated with both age (*r* = .525) and hearing loss (*r* = .615 or higher) as was spatial release (*r* = −.402 or lower for both age and hearing loss). Age and hearing loss were significantly related, however (*r* = .510 or higher), suggesting that multiple regression would be a more appropriate method of exploring the relative influence of age and hearing loss on performance as this would allow the relative contribution of each to be estimated using the same equation.

**Table 1.** Mean thresholds in target-to-masker ratio and the difference, expressed as spatial release from masking (SRM) for each condition in each of the two runs and for the average of both runs.

Condition	Run	*M* (dB)	*SD*
Colocated	1	2.34	1.42
	2	1.57	1.52
	Average	1.96	1.04
Separated	1	−3.29	2.57
	2	−4.72	1.89
	Average	−4.01	1.86
SRM	1	5.63	2.71
	2	6.29	2.19
	Average	5.96	1.83

*Note.* Standard deviations are included.

**Table 2.** Shows the correlations of the SR2 thresholds with age and hearing loss defined as the speech reception threshold (SRT) and pure-tone average, standard (PTA_ST) and high-frequency (PTA_HF).

Predictor variables	Age	Colocated	Separated	Spatial release
Age		**.388**	**.525**	**−.402**
SRT	**.580**	.174	**.646**	**−.641**
PTA_ST	**.510**	.157	**.615**	**−.621**
PTA_HF	**.720**	*.274*	**.627**	**−.576**

*Note.* Values shown in italics are significant at a value of *p* < .05, whereas those in bold are significant at *p* < .001. SR2 = spatial release with two maskers.

As the data were collected over a range of test–retest delays, the number of days between tests (“retest delay”) was also considered and is available as an additional variable in the Supplemental Material S1. There were significant correlations between retest delay and the difference in scores for the colocated condition (*r* = −.304, *p* = .006) and for SRM (*r* = .288, *p* = .009) but not for the separated condition (*r* = .150, *p* = .179). The effects were in the direction of reduced performance with longer delays, as would be expected. The effect sizes were small, however, with regression functions predicting increases between 0.01 dB and 0.03 dB per week of delay.

**Figure 2.**
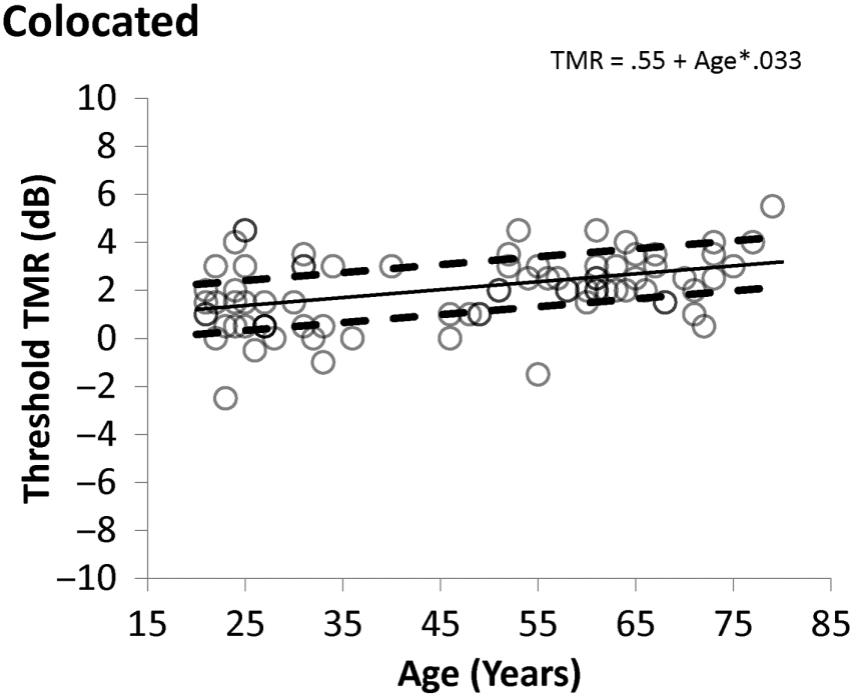
Colocated thresholds as a function of age. The values predicted for the normative function are shown as the solid line and individual thresholds are shown as unfilled circles. Dashed lines indicate the values 1 *SD* away from the predictive function, as defined by the error of the model in predicting the individual data. TMR = target-to-masker ratio.

The data from the average of the two runs are plotted in Figures 2 [Fig F3]–4, along with the predictions of the normative functions calculated from the multiple regression, which was used to examine the effects of age, hearing loss, and the interaction of hearing loss and age on thresholds and SRM. Retest delay was not a significant predictor of performance and so was not included in the analysis. Predicted values and errors of the model for each participant are included in Supplemental Material S1. Plots of the observed and predicted values were examined to ensure linearity, and the errors were examined to ensure a normal distribution. It was clear that linearity and normality were met for the participants with PTA values of 20 dB and better, but the fact that fewer than 20 participants presented with PTAs greater than 20 dB (see Supplemental Material S1) made it difficult to confirm or deny that the assumptions were met for those with greater hearing loss. In the absence of sufficient data to choose an alternative analysis, the linear regression was used. It is noted that future work should both examine a greater sample of those with greater hearing loss and more participants under age 50 years with greater hearing loss, and consider the possibility that nonlinear functions would be a more appropriate approach. The current data set does not allow us to reject the assumption that the linear approach is most appropriate as the predictions are quite accurate for the majority of the participants, as discussed below.

**Figure 3. F3:**
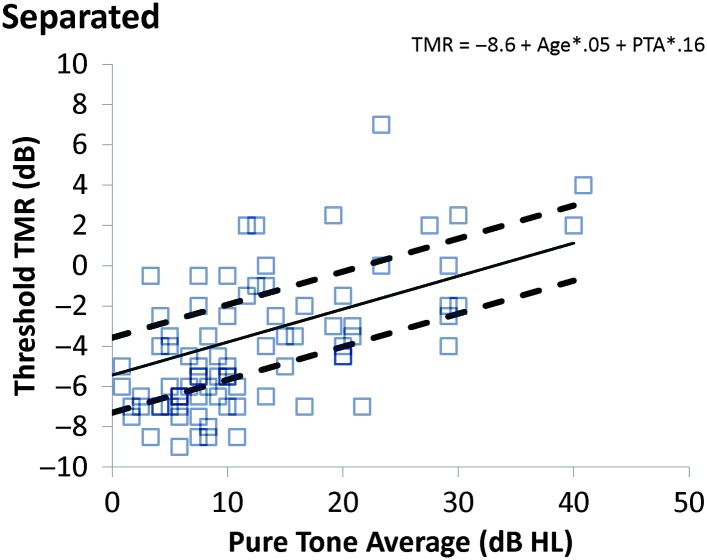
Separated thresholds as a function of pure-tone average (PTA) for the frequencies 0.5, 1, 2, and 4 kHz. The lines and symbols are defined as in Figure 2, and the normative function is calculated with a fixed age of 50 years. TMR = target-to-masker ratio.

**Figure 4.**
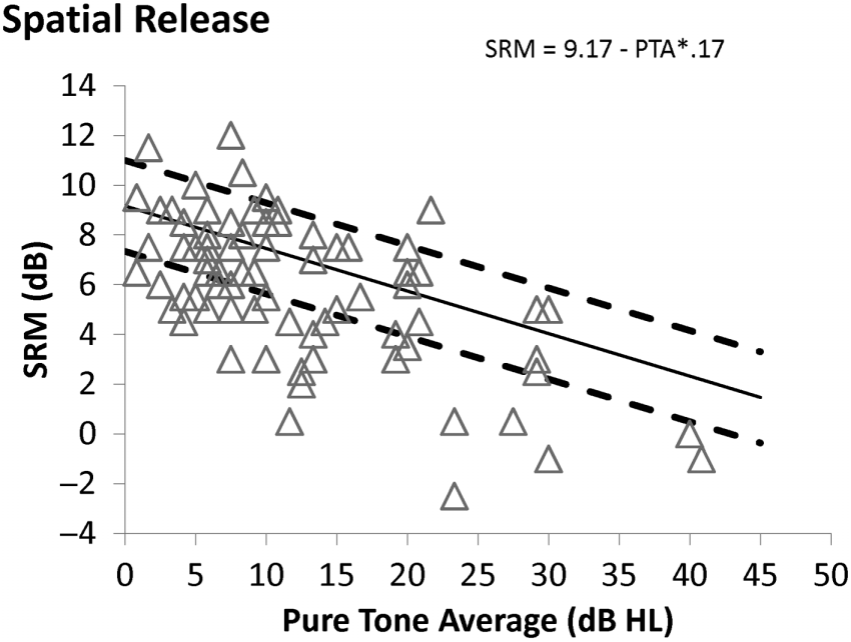
Spatial release from masking as a function of pure-tone average (PTA) for the frequencies 0.5, 1, 2, and 4 kHz. The lines and symbols are defined as in Figures 2 and 3. SRM = spatial release from masking.

Based on the correlations shown in Table 2, standard PTA was used as the measure of hearing loss, as it had the lowest correlation with age. Models were fit for each of the two runs and for the average of the two runs. The results are shown in Table 3, where it can be seen that the basic pattern of results was very similar regardless of whether the data sets being modeled were collected just on the first run, just on the second run, or were an average of the two. The interaction term never accounted for a significant proportion of the variance and so is not shown in the table or included in the normative functions. In the colocated condition, there was a significant effect of age but not of PTA, and the model accounted for only 5%–12% of the variance. In the separated condition, age and PTA were both significant predictors, and the model accounted for 28%–42% of the variance. For SRM, PTA reached significance (23%–37% of the variance). In all conditions, the predictors accounted for more variance in the analysis of the average than in the analysis of either run alone. Based on the power analysis, it was concluded that even the very small relationships were likely to be reliable, especially given the finding that the *p* values were below *p* < .05 for all but one of the colocated conditions (Run 2 had a *p* value of .07) and the regression coefficients were all quite similar. This similarity of the regression coefficients is consistent with the high test–retest reliability found by Jakien et al. (2017) for a larger number of runs with a subset of these same participants.

**Table 3.** Multiple linear regression examining the effects of age and pure-tone average (PTA) on spatial release from masking (SRM) for each of the two runs and for the average of both runs.

Condition	Run	Adjusted *R* ^2^	*p*	Constant	β (age)	β (PTA)	Model error (dB)
Colocated	1	0.071	0.033	0.957	0.035	NS	1.425
	2	0.050	0.070	0.141	0.030	NS	1.525
	Average	0.121	0.005	0.549	0.033	NS	1.045
							
Separated	1	0.280	< 0.001	−8.207	0.058	0.174	2.566
	2	0.406	< 0.001	−9.027	0.047	0.153	1.894
	Average	0.419	< 0.001	−8.617	0.053	0.164	1.864
							
SRM	1	0.226	< 0.001	9.163	NS	−0.188	2.708
	2	0.241	< 0.001	9.168	NS	−0.154	2.189
	Average	0.377	< 0.001	9.166	NS	−0.171	1.830

*Note.* NS = Not Significant; meaning that the predictor was not statistically significant.

Model error was calculated for each participant as the difference between the predicted and the observed value. As can be seen in Table 3, the average value was less than 3.5 dB for all conditions and less than 2.5 dB for the average of two runs. These model error values can be taken as an estimate of the accuracy of the model estimates. These values can be used to transform raw scores for an individual listener into normalized Z-scores as described below.

Figure 2 shows the relationship between age and colocated performance (expressed as estimated TMR threshold), with the solid line indicating the prediction of the regression model for a fixed PTA of 10 dB (although the model predictions are constant across varying PTAs) and the two dashed lines marking 1 *SD* above and below the mean, calculated by treating the average model error in dB as 1 *SD*. Figure 3 shows the relationship between PTA and estimated TMR threshold in the separated condition, with lines indicating the model predictions as PTA varies for a fixed age of 50 years. Figure 4 shows how SRM varies with PTA and the lines show the model predictions for PTA that is varying and age fixed at 50 years (although the model predictions do not depend on age). The equations of the model predictions can be constructed from the values shown in Table 3 by substituting the β values and constant values into Equation 1:

$$\mathrm{Predicted}\;TMR\;\mathrm{threshold}=\mathrm{constant}+\left(\mathrm{age}*\beta \left(\mathrm{Age}\right)\right)+\left(PTA*\beta \left(PTA\right)\right)$$ (1)

From the data shown in Figures 2 –4, it can be seen that some of the participants fall outside the dashed lines. Fewer performed remarkably well than remarkably poorly, probably due to the inability of the progressive method to produce estimated thresholds below −10 dB. To examine the characteristics of those who fell outside the standard interval, the TMR estimates in the SRM condition for the two runs were averaged, and using the values from the model for the average scores in Table 3, each average TMR was converted to a Z-score, using Equation 2, where the predicted TMR comes from Equation 1 and, as mentioned above, the *SD* value is the model error shown in Table 3.

$$Z-\mathrm{Score}=\left(\mathrm{observed}\;TMR-\mathrm{predicted}\;TMR\right)/SD$$ (2)

Seven individuals were observed to have Z-Scores on any of the nine measures (the three conditions, analyzed as individual runs or the average of the two runs), which placed them at or beyond 2 *SD*s from the predicted value for their ages and PTAs. These data are indicated with colored highlighting in the Supplemental Material S1. The records of these individuals were examined to see what factors may be predictors of poor SRM abilities. The age range of the participants was 24–74 years. Three of the participants had normal audiometric thresholds in both ears, whereas four had hearing loss in one or both ears. There was no indication that the presence of tinnitus, a history of concussion, or asymmetrical hearing loss was associated with abnormal performance. Furthermore, only one individual performed abnormally on both runs of either condition. This pattern is further evidence of the strength of the linear model and the accuracy with which the statistical description matches the observed data.

## Discussion

This work describes the creation of normative functions for an automated SRM task, the SR2. The data can be applied to find Z-scores for individuals from 18–80 years of age and with PTAs of less than 40 dB HL. We examined SR2 thresholds for Runs 1 and 2 separately and the average of both. The results of multiple linear regression were similar for all three analyses and revealed (a) a significant effect of age in the colocated conditions, (b) that age and PTA were both significant predictors in the separated conditions, and (c) that for SRM, PTA was the only significant predictor. Consequently, to calculate a Z-score for the colocated condition, only the age of the participant would be needed, and for SRM, only PTA would be needed. For the separated condition, both age and PTA would need to be taken into account to generate a Z-score. The patterns found, which are consistent with those seen by Jakien et al. (2017) for eight runs from a subset of the listeners reported here, are also consistent with the idea that the effects of age are more apparent for small separations while the effects of hearing loss begin to dominate at larger separations. For example, Srinivasan et al. (2016) used a range of spatial separations between target and maskers varying from 0° to 30°and found that, at the smallest separations (up to 6°), there was a significant effect of age on thresholds, whereas at larger separations, the age effect disappeared, and PTA became significant. Other researchers have found age effects in a CRM task, such as Marrone et al. (2008b) and Gallun et al. (2013). The same researchers also found an effect of hearing loss on SRM.

The results reported here are consistent with the data that our laboratory and those of our collaborators have produced using the SR2 and the earlier measures from which the SR2 was derived (e.g., Gallun et al., 2013; Jakien et al., 2017; Marrone et al., 2008b; Srinivasan et al., 2016). In particular, we find again that the effects of hearing loss are more influential than age on performance in the spatially separated condition and that age has similar effects on both the colocated and separated conditions. Hearing loss can act to reduce binaural sensitivity, as many have shown (most recently, e.g., Papesh, Folmer, & Gallun, 2017), but it can also act to reduce the audibility of speech cues. For example, Best et al. (2012) have shown that which speech tasks are used influences SRM and that the differences in performance found in the separated condition reflect both the audibility of the target and the ability of the listeners to use the various cues available to distinguish among the talkers.

The finding that age affects both the colocated condition and the separated condition and has no influence on SRM suggests that there is a small but reliable effect of age on the ability to distinguish among the talkers, regardless of the spatial cues. Perhaps, this is due to a cognitive processing ability that is more likely to be reduced in older participants, or perhaps, it is due to a reduction in sensitivity to fine timing differences in the voices of the male talkers.

By calculating Z-scores, performance of individual research participants and patients can be examined in relation to the expected values, reducing the costs and effort needed to run a control group to compare to an experimental group and allowing individual patients to be characterized with reference to the expected performance for an individual of their age and hearing ability. This would have been especially helpful for those studies that, due to availability of patients and/or resources, have examined small samples of participants on the SR2. For example, were normative values available, it would perhaps have been possible to draw stronger conclusions about the influence of brain injury on SRM as measured by the SR2 (Hoover, Souza, & Gallun, 2017). Similarly, normative functions would have allowed Papesh et al. (2017) to transform their thresholds in the SR2 into Z-scores prior to the regression analysis in which reliable relationships were revealed between electrophysiological measures of binaural sensitivity and performance on the SR2. Without such normative functions, it is difficult to tell whether age and hearing loss were reducing binaural sensitivity and also reducing performance on the SR2 or whether it was truly the reduced binaural sensitivity that led to the reduced performance on the speech tasks.

Limitations of this study include the age range of the participants, which did not include children or listeners older than 80 years, and hearing loss, which did not exceed PTAs of 45 dB HL. It is likely that the linear functions would not predict data including children due to the poorer performance of the younger children relative to the teenagers and young adults. Similarly, it is not clear whether the participants older than 80 years would be as well predicted as their younger counterparts. These tools should be used to explore these populations in the future. Hearing loss was limited in order to ensure that equal audibility would be available to all participants, but this means that the functions are most clearly valid for listeners with hearing in the normal range. As the predicted spatial release diminishes to near 0 at 50 dB HL, however, this limitation is less a characteristic of the sample and more related to the nature of spatial release. Future work should (a) examine those with greater hearing loss, however, as only four of the eight participants with thresholds above 25 dB HL achieved spatial release near the predicted values (see Figure 4) and (b) involve psychophysical and cognitive measures as covariates. The availability of normative functions will allow such studies to be conducted using a smaller group of participants who are tested repeatedly on a range of measures, which will increase the power of the analysis.

## Summary

Translation of laboratory tests into a form suitable for clinical testing requires the transformation from tests that function well for comparing groups of participants to tests that can be used to evaluate a single individual. With the normative functions generated from this data set, it would be possible for a researcher or clinician to interpret data from a small number of participants or even a single patient without having to first collect data from a control group, substantially reducing the time and resources needed.

## Supplementary Material

10.1044/2018_AJA-17-0069SMS1 Supplemental Material S1. The full audiogram data for each participant, along with gender, hearing aid use, and calculated values of PTA and asymmetry across ears.
